# An ecological approach to neural computation

**DOI:** 10.1186/1471-2202-14-S1-P40

**Published:** 2013-07-08

**Authors:** Romain Brette

**Affiliations:** 1 Institut d'Études Cognitives, École Normale Supérieure, Paris, 75005, France; 2 Laboratoire de Psychologie de la Perception, CNRS and Université Paris Descartes, Paris, 75006, France

## 

In traditional theories of perception, primitives are extracted from sensory signals, and ecologically relevant stimuli are described in terms of complex combinations of these primitives. Recognizing these combinations requires learning. James Gibson criticized this view, for the concept of primitive is a mathematical abstraction that may have no ecological meaning. What is elementary for a sensory system may not be simple in terms of mathematical primitives, and conversely. Instead, Gibson argued that objects in an animal's environment produce sensory signals that are highly structured, and the invariant structure is what is meaningful about the environment. He proposed that sensory systems directly extract this invariant structure.

I will address two questions, focusing on the auditory system: 1) what invariant structure can be considered elementary for a sensory system? 2) how can the nervous system extract this structure?

I define "elementary structure" as the set of identities between the time-varying sensory signals produced by different receptors, up to a delay. That is, S_i_(t) = S_j_(t+T), where i and j are receptor indexes. In the cochlea, this corresponds to a regularity structure in the spatio-temporal pattern of vibration of the basilar membrane.

In a monaural context, sounds that produce such an elementary structure are periodic sounds, that is, sounds that elicit a pitch percept. I postulate that the pitch of a sound is precisely the elementary structure that it produces. When taking into account the physiological constraints in the identification of this structure (essentially, that conduction delays are limited), it explains many phenomena that were previously unexplained by any single model: lower and upper limits of pitch, the distinction between resolved and unresolved harmonic complexes, the topology of pitch (e.g. the octave similarity), and slight level dependences of pitch.

In a binaural context, stimuli that produce an elementary structure across the two cochleas are binaural sounds produced by a single source in the environment. The elementary structure depends on source location but not on the signal emitted by the source. This structure is much more complex, mathematically, than often described. In particular, the interaural time difference (ITD) depends on frequency. I will show evidence from single unit recordings in the inferior colliculus of cats that binaural neurons do indeed encode this structure, and not the mathematically simpler ITD.

How can this structure be extracted by the nervous system? Since neurons are highly sensitive to coincident inputs, a natural question to ask is for what signals the presynaptic neurons produce synchronous spikes. This yields to the concept of the *synchrony receptive field *of a group of neurons, defined as the set of stimuli that elicit synchronous responses in these neurons. When applied to sensory neurons, the synchrony receptive field is precisely the elementary structure of sensory signals, and it can be decoded by postsynaptic neurons by coincidence detection. I will demonstrate that simple spiking models based on these ideas can indeed decode the elementary structure of auditory signals corresponding to pitch and sound location. This structure is elementary from the environmental viewpoint, but not from the mathematical viewpoint. Therefore, I suggest that this approach provides simple practical solutions to non-trivial sensory problems.
